# Exploratory Metabolomics Underscores the Folate Enzyme ALDH1L1 as a Regulator of Glycine and Methylation Reactions

**DOI:** 10.3390/molecules27238394

**Published:** 2022-12-01

**Authors:** Blake R. Rushing, Halle M. Fogle, Jaspreet Sharma, Mikyoung You, Jonathan P. McCormac, Sabrina Molina, Susan Sumner, Natalia I. Krupenko, Sergey A. Krupenko

**Affiliations:** 1 Nutrition Research Institute, UNC Chapel Hill, Kannapolis, NC 28081, USA; 2 Department of Nutrition, UNC Chapel Hill, Chapel Hill, NC 27599, USA

**Keywords:** folate, ALDH1L1, cancer, metabolomics

## Abstract

Folate (vitamin B9) is involved in one-carbon transfer reactions and plays a significant role in nucleic acid synthesis and control of cellular proliferation, among other key cellular processes. It is now recognized that the role of folates in different stages of carcinogenesis is complex, and more research is needed to understand how folate reactions become dysregulated in cancers and the metabolic consequences that occur as a result. ALDH1L1 (cytosolic 10-formyltetrahydrofolate dehydrogenase), an enzyme of folate metabolism expressed in many tissues, is ubiquitously downregulated in cancers and is not expressed in cancer cell lines. The RT4 cell line (derived from papillary bladder cancer) which expresses high levels of ALDH1L1 represents an exception, providing an opportunity to explore the metabolic consequences of the loss of this enzyme. We have downregulated this protein in RT4 cells (shRNA driven knockdown or CRISPR driven knockout) and compared metabolomes of ALDH1L1-expressing and -deficient cells to determine if metabolic changes linked to the loss of this enzyme might provide proliferative and/or survival advantages for cancer cells. In this study, cell extracts were analyzed using Ultra High Performance Liquid Chromatography High Resolution Mass Spectrometry (UHPLC-HR-MS). A total of 13,339 signals were identified or annotated using an in-house library and public databases. Supervised and unsupervised multivariate analysis revealed metabolic differences between RT4 cells and ALDH1L1-deficient clones. Glycine (8-fold decrease) and metabolites derived from S-adenosylmethionine utilizing pathways were significantly decreased in the ALDH1L1-deficient clones, compared with RT4 cells. Other changes linked to ALDH1L1 downregulation include decreased levels of amino acids, Krebs cycle intermediates, and ribose-5-phosphate, and increased nicotinic acid. While the ALDH1L1-catalyzed reaction is directly linked to glycine biosynthesis and methyl group flux, its overall effect on cellular metabolism extends beyond immediate metabolic pathways controlled by this enzyme.

## 1. Introduction

**Figure 1:**
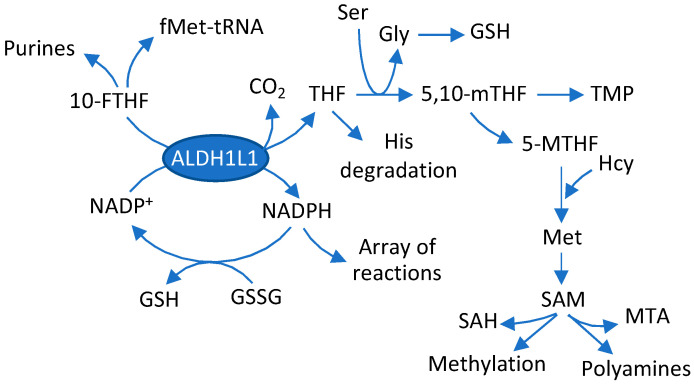
ALDH1L1-catalyzed reaction and downstream metabolic pathways. The immediate downstream processes linked to ALDH1L1 are: biosynthesis of purine nucleotides; formylation of Met-tRNA (the reaction required for the initiation of protein biosynthesis in mitochondria); the generation of THF; and the NADPH production. THF is required for glycine biosynthesis from serine and histidine degradation. Downstream of glycine synthesis are shown GSH, TMP and SAM biosynthesis; SAM is linked to the variety of methylation reactions and polyamine biosynthesis. The role of NADP^+^/NADPH is widespread in the cell; as the example, the NADPH-dependent reduction of oxidized glutathione (GSSG) is shown. GSH, reduced glutathione; THF, tetrahydrofolate; 10-FTHF, 10-formyl-THF; 5,10-mTHF, 5,10-methylene-THF; 5-MTHF, 5-methyl-THF; TMP, thymidine monophosphate; SAM, S-adenosylmethionine.

ALDH1L1 (cytosolic 10-formyltetrahydrofolate dehydrogenase) is an abundant enzyme in the liver, pancreas, and several other tissues [1]. This enzyme catalyzes the conversion of 10-formyltetrahydrofolate to tetrahydrofolate (THF) and CO_2_ while simultaneously reducing NADP^+^ to NADPH [2]. This reaction regulates the availability of one-carbon groups (OCG) from the reduced folate pool for biosynthetic reactions [3]. Such a role is based on the phenomenon that OCG converted to CO_2_ leave the pool and cannot be used in folate-associated biochemical reactions. It is also believed that the ALDH1L1 reaction replenishes the THF pool, which is required to accept OCG in the reactions of glycine biosynthesis from serine, glycine and histidine degradation, and formate utilization [2]. While previous research confirmed the role of ALDH1L1 in the regulation of folate-bound OCG [4,5], several recent studies revealed its role in the regulation of glycine biosynthesis and histidine degradation [6,7,8]. Thus, the knockout of *Aldh1l1* in mice causes a significant drop in glycine levels in liver and plasma as well as decreased levels of glycine conjugates. FIGLU (formiminoglutamate), the marker of folate deficiency, was highly elevated in the KO mice [6]. FIGLU is an intermediate in the folate-dependent histidine degradation and it accumulates if there is no sufficient THF to further metabolize this compound, which is typically associated with folate deficiency [9,10]. In the case of the ALDH1L1 loss, such an effect is rather associated with insufficiency of THF while the overall folate levels are not strongly changed [6]. Overall, the ALDH1L1-catalyzed reaction is linked to many metabolic processes in the cell (Figure 1).

Numerous studies also demonstrated that ALDH1L1 is strongly and ubiquitously downregulated in different types of human cancers [1,11,12,13,14,15]. Thus, it has been recognized as the most under expressed protein in hepatocellular carcinoma and in metastatic liver tumors [12]. ALDH1L1 downregulation in cancers was linked to hypermethylation of the large CpG island in the gene promoter [16]. Differential ALDH1L1 methylation was shown in lung, breast, cervical, kidney, and colon cancers [11,14,17,18]. As well, cancer cell lines do not express ALDH1L1 and present strong hypermethylation of the ALDH1L1 promoter region [1,5,16,19]. Re-expression of ALDH1L1 through transient or induced stable transfection in cancer cells typically leads to inhibition of proliferation and the induction of apoptosis [1,5,19,20,21]. We have recently found a cancer cell line, RT4, expressing high levels of ALDH1L1. These cells were derived from a grade I urothelial carcinoma and they represent a low-grade bladder cancer [22,23,24]. Our previous [25] experiments confirmed presence of high levels of ALDH1L1 in these cells. In the present study, we knocked out ALDH1L1 in RT4 cells using shRNA and CRISPR and compared metabolomes of ALDH1L1-expressing and -deficient cells to address the question of what metabolic changes linked to the loss of this enzyme might provide proliferative and survival advantages for cancer cells.

## 2. Results

### 2.1. Knockout of ALDH1L1 in RT4 Cells

**Figure 2:**
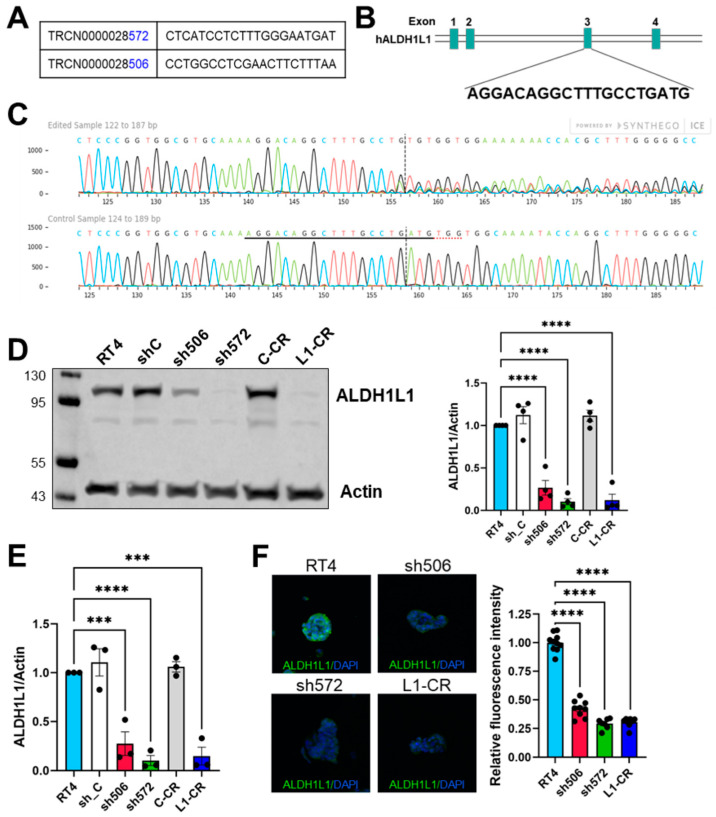
(**A**) shRNA targeting sequences of ALDH1L1 gene, (**B**) ALDH1L1 sequence in exon 3 targeted by CRISPR/Cas9, (**C**) genomic sequencing of the RT4 clone L1-CR and control RT4 cells confirms successful targeting ALDH1L1, (**D**) ALDH1L1 protein levels (**left** panel) and bands quantification (**right** panel), (**E**) Distribution of ALDH1L1 mRNA levels, (**F**) Immunofluorescence staining of ALDH1L1; plot shows quantification of green fluorescence (ALDH1L1) using Fiji-Image J (NIH). Apparent residual fluorescence in clones 572 and L1-CR represents background. Multigroup comparisons were performed by a one-way ANOVA with Dunnett’s multiple comparisons using GraphPad Prism 9. **** *p* < 0.0001; *** *p* < 0.001.

To evaluate the effect of ALDH1L1 loss in RT4 cells, we targeted its expression by shRNA as well as by CRISPR knockout. A total of five shRNA with different target sequences were used. Two clones, 506 and 572, generated using shRNA (Figure 2A) and one clone, L1-CR, generated by CRISPR (Figure 2B,C), were analyzed. Western blot assays have shown that clones 572 and L1-CR completely lost ALDH1L1 expression (Figure 2D, ALDH1L1 in these clones is below the detection limit). Clone 506 still expressed ALDH1L1, but its protein levels were about 2.5-fold lower than in original RT4 cells (Figure 2D). Levels of ALDH1L1 mRNA evaluated by qPCR were also strongly downregulated in all three clones (Figure 2E). The loss of ALDH1L1 in all three clones was further confirmed by immunocytochemical staining with an ALDH1L1-specific antibody (Figure 2F).

### 2.2. Overall Metabolomic Analysis

**Figure 3:**
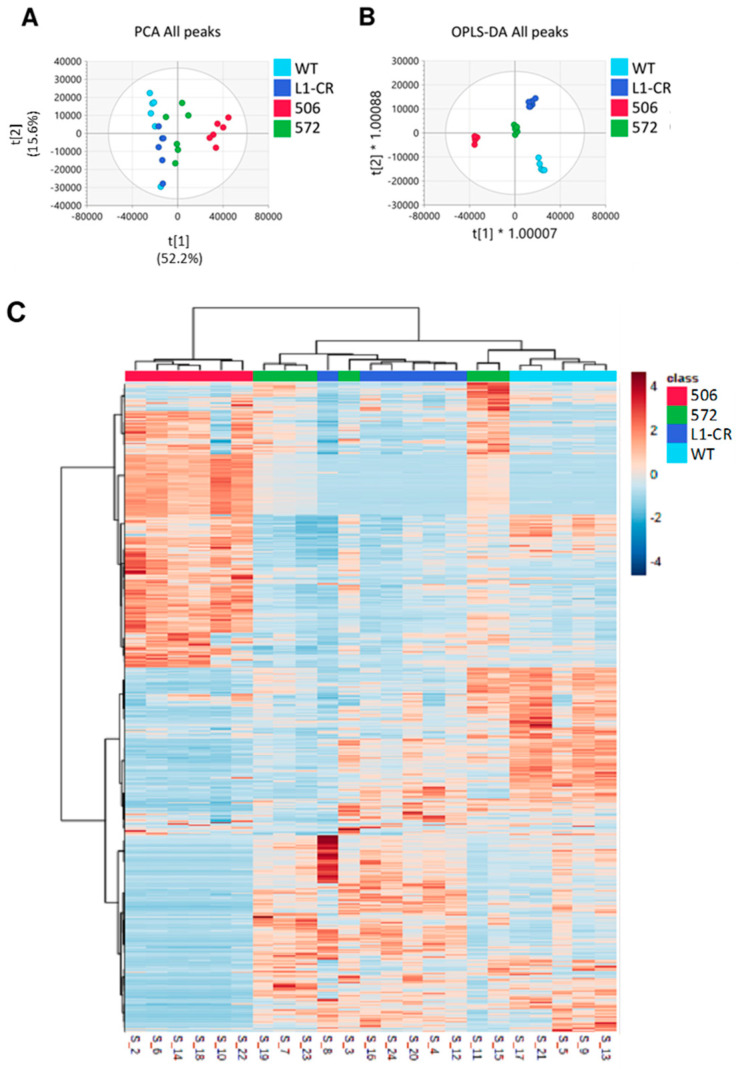
Comparison of RT4 cells and ALDH1L1-deficient clones based on all peaks from untargeted metabolomics data. PCA (**A**) and OPLS-DA (**B**) between all groups with the CRISPR and 572 clones clustered closely (R2X: 0.920, R2Y: 0.981, Q2: 0.808). (**C**) A heat map (generated using MetaboAnalyst 5.0 [26]) of measured metabolites (13,339 total, Supplementary File S1) demonstrates significant differences between groups’ metabotypes, with WT RT4 cells and clone 506 being most distant and CRISPR and 572 clones being the farthest apart. The heatmap is auto-scaled (mean-centered and divided by standard deviation) for each variable; n = 5 per group (RT4 cells) and 6 per groups for each clone. Orange colors represent higher auto-scaled values whereas blue colors represent lower auto-scaled values. Colors for experimental groups are as follows: 506, red; 572, green; L1-CR, dark blue; WT, cyan. Hierarchical clustering was performed on samples in MetaboAnalyst 5.0 using Euclidean distance measures. Each sample number (as in Supplementary File S1) is indicated at the bottom of the heatmap.

We have performed untargeted metabolomics analysis of RT4 cells and the three clones, 506, 572, and L1-CR. A total of 25,373 peaks remained after preprocessing and 13,339 metabolites were identified and annotated, with 259 metabolites assigned with OL1 and OL2a confidence and an additional 587 metabolites matched as OL2b and PDa. Supervised and unsupervised analysis using all peaks showed good differentiation between study groups (Figure 3A,B and Supplementary Figure S1). PCA performed using data for metabolites that matched to the in-house physical standards library were visually similar to that conducted using all signals (Supplementary Figure S1). The first component of PCA showed the most spatial distance between clone 506 and original RT4 cells, with 572 and L1-CR groups being less different from RT4 cells (Figure 3A,B and Supplementary Figure S1). This analysis also indicated that clones 572 and L1-CR have close metabolic profiles. Pairwise supervised analysis (OPLS-DA) was used as an additional method to evaluate differences between the RT4 cell group and each of three clones (Supplementary Table S1), which is further illustrated by generated heatmaps (Figure 3C).

### 2.3. Top Metabolites Separating RT4 Cells and ALDH1L1-Deficient Clones

**Figure 4:**
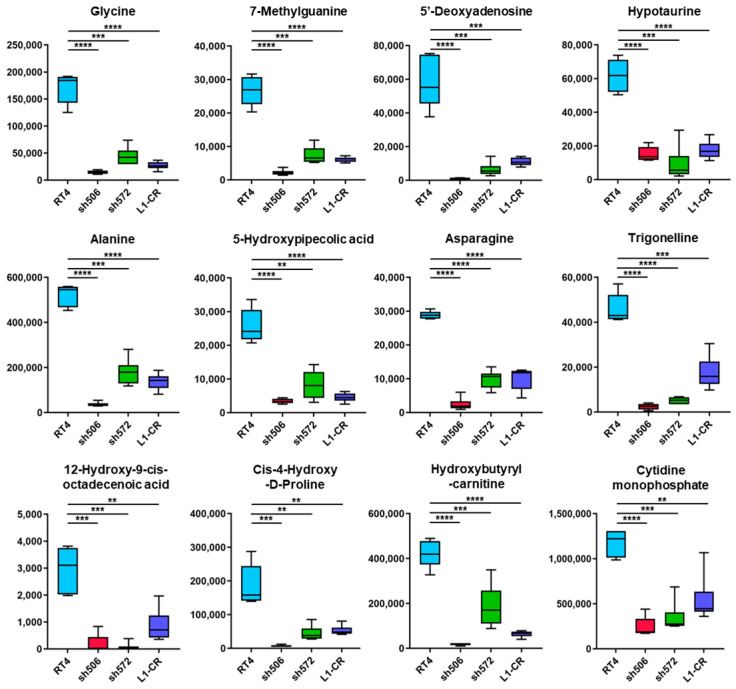
Boxplots showing the distribution of normalized peak area counts from the MS analysis for top significant metabolites (OL1 and OL2a ontology levels) based on the volcano plot in Supplementary Figure S2B that differentiate RT4 cells and three ALDH1L1 depleted clones. The values on the y axis represent normalized peak area counts. FDR-corrected *p* values are: **** *p* < 0.0001; *** *p* < 0.001; ** *p* < 0.01. Non-corrected and Bonferroni-corrected *p* values for the pairwise comparison of RT4 cells and each clone are shown in Supplementary Table S2 (metabolites from Figure 4 are highlighted in the table).

The top metabolite significantly decreased in all three ALDH1L1-deficient clones was glycine (Figure 4). Six other amino acids (serine, asparagine, alanine, threonine, glutamate, and proline) were also affected in all three clones in a similar way (Figure 4, Supplementary Table S2 and Supplementary File S1). Two other top metabolites decreased in all clones were 7-methylguanine and 5′-deoxyadenosine (Figure 4 and Supplementary Table S2). Additional metabolites strongly affected by the loss of ALDH1L1 include nucleotides, polyamines, citric acid cycle intermediates, and carnitine/acyl carnitines (Supplementary Table S2). To identify metabolites which commonly and most strongly changed in response to ALDH1L1 downregulation, we further combined samples from all three clones in a single group and compared this group with wildtype RT4 cells using OPLS-DA and univariate analyses (Supplementary Figure S2A and Supplementary File S2). Peak abundances of identified metabolites (OL1 and OL2a ontology levels, total of 259 metabolites) were uploaded to MetaboAnalyst 5.0 for statistical analysis, and a volcano plot was built to determine metabolites showing most significant difference between wild-type and all L1-knockout clones combined in one group (Supplementary Figure S2B). The total number of metabolites significantly changed (*p* < 0.05) in ALDH1L1-deficient clones were 97 (91 decreased/6 increased), 87 (86/1) or 60 (60/0) for non-corrected, FDR-corrected and Bonferroni corrected p values, correspondingly (Supplementary Table S3). Of note, metabolites shown in Figure 4 were highly significant in this analysis as well, with glycine being the most significant (Supplementary Table S3, highlighted).

### 2.4. Metabolic Differences between RT4 Cells with High, Low, and Undetectable Levels of ALDH1l1

**Figure 5:**
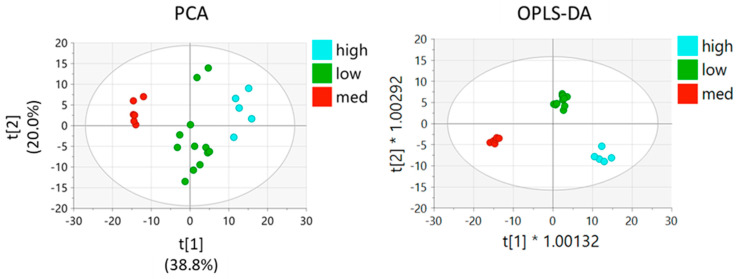
Analysis of metabolomics data (PCA and OPLS-DA) based on the ALDH1L1 expression levels in three-groups (WT RT4 cells with high levels of ALDH1L1, high group; clone 506 with intermediate levels of ALDH1L1, medium group; L1-CR and 572 clones, low/undetectable ALDH1L1, low group).

**Figure 6:**
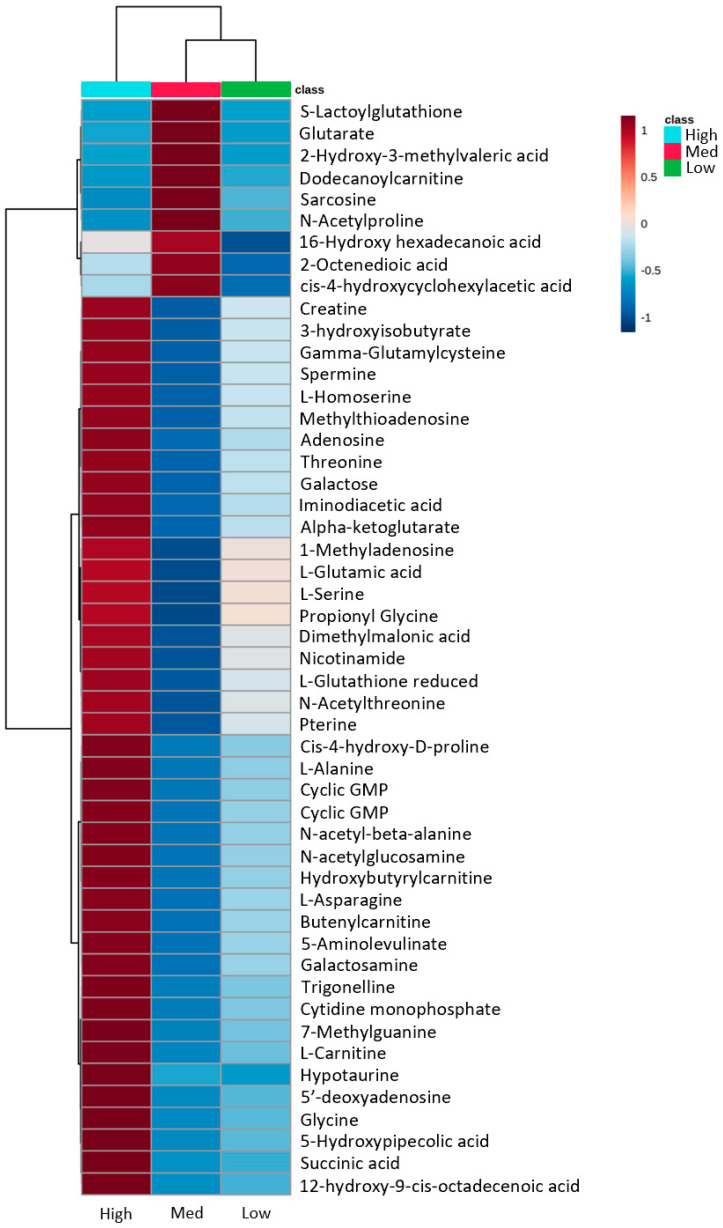
The heat map generated using OL1 and OL2a metabolites to visualize differences between groups in the three-group analysis. The heatmap is auto-scaled (mean-centered and divided by standard deviation) for each variable; n = 5 (high ALDH1L1, RT4 cells, cyan); n = 6 (medium ALDH1L1, clone 506, red); n = 12 (low/undetectable ALDH1L1, L1-CR and 572 clones, green). Orange colors represent higher auto-scaled values whereas blue colors represent lower auto-scaled values. Hierarchical clustering was performed on samples in MetaboAnalyst 5.0 using Euclidean distance measures.

Our study showed that clone 506 expresses detectable, though much lower than original RT4 cells, levels of ALDH1L1 while such levels were almost undetectable at the protein level in 572 and L1-CR clones (Figure 2D). Because PCA and OPLS-DA of untargeted metabolomic data revealed that the two clones lacking ALDH1L1 are clustered together and are well separated from RT4 cells and the 506 clone, we performed further analysis comparing three groups based on the ALDH1L1 expression (high, medium, and low/no expression). PCA as well as supervised analysis (OPLS-DA) showed clustering of samples for each of the three groups (Figure 5), which is also illustrated by the heatmap in Figure 6. Intriguingly, this analysis showed that many metabolites display the U-shaped relationship with ALDH1L1 levels (Supplementary Figure S3). The volcano plots and corresponding lists of metabolites discriminating the groups in pairwise analysis are shown in Supplementary Figure S4 and Supplementary File S3, correspondingly.

### 2.5. Construction of ALDH1L1-Dependent Metabolic Network

**Figure 7:**
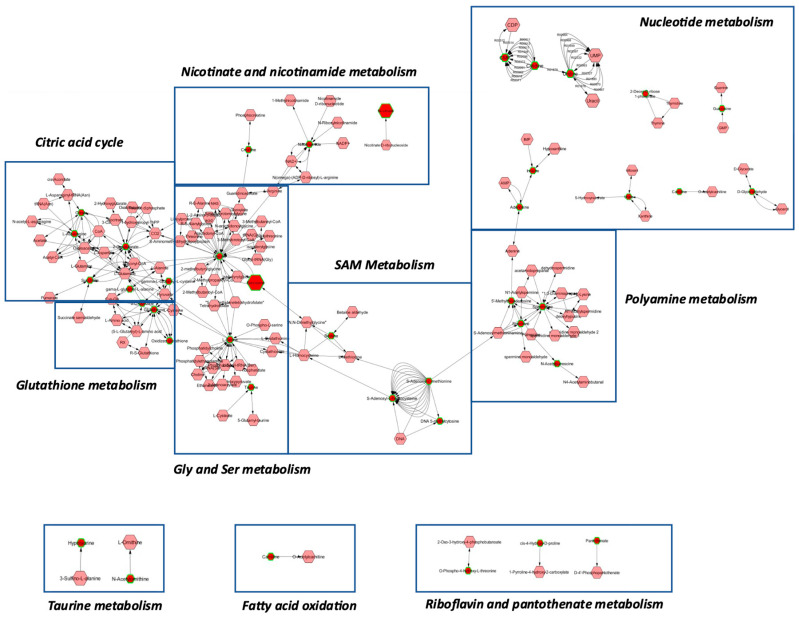
ALDH1L1-dependent metabolic network constructed based on comparison between WT group and combined group of ALDH1L1 targeted cells. OL1 and OL2a level metabolites were uploaded to Metscape with p-values and fold changes. Metabolites were organized into pathways using the KEGG human database. Dark red nodes with green outline are metabolites significantly different between WT and all KO groups (FDR-corrected *p* < 0.05). Light red nodes are metabolites included in the KEGG database for these pathways, which differences were not significant between WT and all KO groups in our experimental data. The size of the nodes indicates the direction of change—small nodes are decreased in KO samples whereas large nodes are increased. Edges represent known enzymatic reactions in the database that connect two nodes.

Metabolites with an OL1 and OL2a ontology level were analyzed to determine metabolic networks associated with the ALDH1L1 loss. For this analysis, all ALDH1L1 knockout clones were combined into one group and compared against wildtype RT4 cells. Our analysis showed that alterations in S-adenosylmethionine (SAM) metabolism were central to ALDH1L1’s metabolic effects, acting as link between perturbations in glycine metabolism, glutathione metabolism, citric acid cycle, nicotinate and nicotinamide metabolism, polyamine metabolism, and nucleotide metabolism (Figure 7). These findings correspond well to the metabolic role of the ALDH1L1 catalysis in the cell (Figure 1). Metabolites in additional pathways including fatty acid metabolism, vitamin B2 and B5 metabolism, and ornithine and taurine metabolism were also affected (Figure 7). These results show that ALDH1L1 displays far-reaching metabolic effects, and the perturbations in methylation reactions are central to these effects. The downstream metabolic effects of ALDH1L1 catalysis could be also associated with the generation of NADPH, the coenzyme involved in numerous pathways in the cell [27]. While the ALDH1L1 reaction is not considered as one of the main routs of NADPH production [28], its mitochondrial homolog ALDH1L2 was shown to significantly contribute to the mitochondrial NADPH pool [29]. Of note, nicotinate and nicotinamide pathways were linked to the ALDH1L1 metabolic network (Figure 7). Pathway analysis of significantly increased or decreased metabolites matched to the in-house library showed significant perturbations in the pathways identified in the network analysis (Supplemental File S4), supporting the conclusion that these pathways are under regulation by ALDH1L1.

## 3. Discussion

A large body of literature indicates that levels of numerous metabolites are very different between cancer and normal cells [30,31,32,33]. This phenomenon is associated with different metabolic requirements of proliferating versus quiescent cells and is supported by significant re-wiring of multiple metabolic pathways [30,34,35,36,37]. Altered metabotype of cancer cells allows unlimited proliferation, provides selective advantage in a hypoxic environment, and enables additional properties such as detachment from the site of origin, enhanced motility, migration, and adhesion [38,39]. Furthermore, metabolic profiles are commonly different between cancers of different stages [40,41]. To reshape metabolic landscape, cancer cells implement diverse changes in expression of enzymes involved in critical pathways with many enzymes being up- and down-regulated [42,43]. However, there is a more limited number of key enzymes, commonly catalyzing rate-limiting steps in pathways, which alterations exhibit more profound effects associated with cancer development [37,44,45,46]. One of these such enzymes might be ALDH1L1, which regulates folate pathways by disposing one-carbon groups as CO_2_ [2,3].

Based on ubiquitous downregulation of ALDH1L1 in human cancers, the protein has been considered as a putative tumor suppressor [1,3]. If this is the case, it would be expected that the loss of this protein benefits proliferation of cancer cells or provides a survival advantage in growing malignant tumors. This hypothesis has found support in our recent study of diethylnitrosamine-induced hepatocellular carcinoma in mice, which indicates that *Aldh1l1* knockout promoted liver tumor growth without affecting tumor initiation or multiplicity [47]. Metabolomic analysis in that study further indicated a strong effect of the ALDH1L1 loss on liver metabolism. We were also interested to explore the direct effect of the enzyme downregulation on metabolic profile of human cancer cells. However, our previous studies of numerous human cancer cell lines did not identify any cancer cells expressing ALDH1L1. This includes the HepG2 cell line [1], which has a relatively high level of ALDH1L1 mRNA, according to The Human Protein Atlas (https://www.proteinatlas.org, accessed on 15 August 2022). Additionally, a report indicating detectable levels of this protein in A549 cells (lung adenocarcinoma) [48] could be a false positive result due to antibody specificity as we discussed in our commentary [25].

The Human Protein Atlas (https://www.proteinatlas.org, accessed on 15 August 2022) shows that from a list of provided cell lines RT4 cells have the highest levels of ALDH1L1 mRNA. The expression of ALDH1L1 in RT4 cells was also confirmed at the protein level [49]. As well, in our previous study we have observed high levels of ALDH1L1 mRNA and protein in RT4 cells [25]. Thus, this cell line offered an opportunity to study the effect of the enzyme knockout on the cellular metabolic profile. In our study, both shRNA and CRISPR-driven ALDH1L1 knockout caused a strong decrease in levels of mRNA and protein. While numerous metabolites and pathways were affected upon ALDH1L1 downregulation, the main one discriminating the ALDH1L1 knockdown clones and original RT4 cells was glycine, levels of which were significantly dropped. This finding is in line with our previous reports regarding the role of the enzyme in glycine regulation [6,7,8,47]. Mechanistically, this is due to the role of the enzyme in the generation of THF, the co-substrate in the reaction of glycine biosynthesis from serine (Figure 1). Of note, glycine was highlighted as a metabolite decreased in bladder cancer [22]. Levels of two other amino acids, alanine and asparagine, were also strongly decreased in ALDH1L1-deficient clones. Interestingly, levels of alanine were different between RT4 cells and more invasive TCCSUP bladder cancer cells, a phenomenon linked to a difference in the pyruvate consumption [50]. Perturbed alanine metabolism was also associated with non-muscle invasive bladder cancer [51]. Curiously, ALDH1L1 was one of the most elevated proteins in RT4 cells treated with benzo[a]pyrene; several amino acids were simultaneously elevated in this study [49]. Although these changes cannot be assigned only to the expression of ALDH1L1, the same amino acids were notably decreased in RT4 clones upon the ALDH1L1 loss in our study. Another top pathway differentiating the metabotype of RT4 cells and its ALDH1L1-depleted clones was glutathione metabolism. Of note, glutathione plays a role in a variety of malignancies (reviewed in [52]), including the contribution of glutathione synthesis to the risk of bladder cancer [53].

Two other metabolites most significantly decreased in ALDH1L1-deficient RT4 clones were 7-methylguanine and 5′-deoxyadenosine. We attributed these changes to the pathways linked to the S-adenosylmethionine (SAM) metabolism. Indeed, decreased ALDH1L1 levels were also associated with the decrease in SAM and several other metabolites originated from SAM-dependent reactions. These metabolites include polyamines, MTA (methylthioadenosine), SAH (S-adenosylhomocysteine) and trigonelline. Trigonelline, or N-methylnicotinic acid, is excreted in the urine [54] and is a product of the metabolism of niacin (vitamin B3) [55]. While metabolism of trigonelline is not well understood, its levels in our study inversely correlated with levels of nicotinic acid, supporting the hypothesis that it is a product of niacin degradation. SAM is the universal methyl donor in the cell, but it is also involved in polyamine biosynthesis where it serves as a donor of the aminopropyl group [56]. In addition, SAM participates in radical reactions, in which its reductive cleavage produces 5′-deoxyadenosine as byproduct [56]. Though only eight radical SAM enzymes are known in humans [56], a strong decrease of 5′-deoxyadenosine suggests a significant decline of this type of catalysis upon ALDH1L1 downregulation.

Curiously, 7-methylguanine has been linked to bladder cancer. Targeted metabolomics has shown that levels of 7-methylguanine in urine are significantly different between patients with bladder cancer and healthy volunteers [57]. Further, the occurrence of 7-methylguanine in DNA from bladder tumors was higher than in adjacent normal bladder epithelium [58]. Methylation of guanine at the N7 position is a very common DNA modification. Its repair includes the excision of 7-methylguanine by N-methylpurine DNA glycosylase leaving abasic sites, which increases probability for DNA strand breaks [59]. Thus, increased levels of 7-methylguanine could be an indicator of enhanced cell death. Furthermore, this compound is a natural inhibitor of the DNA repair enzyme PARP and by itself may exhibit an anticancer activity [60]. Considering these two mechanisms, decreased levels of 7-methylguanine in ALDH1L1-deficient clones could be indicative of a better cell survival. Of note, levels of 7-methylguanine in normal colorectal mucosa biopsies inversely correlated with folate consumption [61].

A recent report indicated that RT4 cells display an activated oxidative phosphorylation, a feature likely associated with the origin of these cells from a low-grade tumor [62]. Indeed, in this study bladder cancer cell line 5637 from a high-grade tumor relied mainly on glycolysis to produce energy. The study concluded that bladder cancer cell lines associated with a low risk of progression present an activated oxidative metabolic state, while those associated with a high risk present a non-oxidative state and high glycolytic activity [62]. In this regard, our metabolomic analysis showed significant decrease in intermediates of the Krebs cycle in ALDH1L1 deficient clones suggesting a metabolic shift relevant to the mitochondrial energy generation. It should be noted though that the link between the tumor grade and energy-related metabolic profiles is more complex. For example, a study of metabotypes of urothelial bladder cancer cell lines of different grades indicated that metabolic pattern for grade I cells (RT4) is more similar to grade IV (more advanced tumors) than grade III [24].

When we analyzed metabolomics data as the three-group comparison (high, medium, and low/undetectable ALDH1L1 protein), a U-shape effect was observed for numerous metabolites with RT4 cells (high ALDH1L1) and two ALDH1L1-deficient clones showing similar trend when compared to clone 506 (medium ALDH1L1 expression). Though the precise basis for such effect is not clear, a previous study has shown that low versus high expression of ALDH1L1 in neuroblastoma cells has a different effect on reduced folate pool: the low expression facilitates the incorporation of one-carbon groups into the pool, whereas high expression depletes such groups from the pool [4].

The expression of ALDH1L1 in two cell lines of non-cancerous origin, HEK293 and NIH3T3, was reported [1,63], indicating that the antiproliferative effect of the enzyme is likely linked to a cancer metabotype. In this regard, it is not clear why, in contrast to most other cancer cell lines, RT4 cells express ALDH1L1 to a significant level. Apparently, mechanisms compensating for the metabolic effect of ALDH1L1 [64] might function in these cells. On the other hand, RT4 cells represent low grade bladder cancer, which might have different metabolic requirements than high-grade cancers. This also raises the question of whether ALDH1L1 can serve as a marker of bladder cancer aggressiveness.

## 4. Materials and Methods

### 4.1. Cell Culture

The human bladder cancer cell line RT4 (ATCC HTB-2) was purchased from American Type Culture Collection (ATCC, Manassas, VA, USA) and were grown in McCoy’s 5A medium (Thermo Fisher Scientific, Waltham, MA, USA) supplemented with 10% fetal bovine serum (FBS, Bio-Techne, Minneapolis, MN, USA) and 1% of antibiotic-antimycotic (Thermo Fisher Scientific) at 37°C in a humidified atmosphere of 5% CO_2_.

### 4.2. Generation of Aldh1l1-Knockout Cell Lines

ALDH1L1-deficient RT4 cells were generated using shRNA or CRISPR/Cas9 techniques. Five lentiviral shRNAs targeting ALDH1L1 (TRCN0000028506; TRCN0000028529; TRCN0000028539; TRCN0000028567; and TRCN0000028572) were purchased from Sigma-Aldrich (MISSION shRNA Lentiviral Transduction Particles, St. Louis, MO, USA). RT4 cells were infected with lentivirus harboring either non-targeting control (MISSION TRC2 pLKO.5-puro Empty Vector Control), or ALDH1L1 targeting shRNA. After 48 h, transduced cells were selected in the medium containing 10 µg/mL puromycin for 3–4 weeks. Once the cells reached optimal growth rates, the polyclonal populations were maintained (multiplicity of infection (MOI) is 10 for each clone). Knockdown efficiency of ALDH1L1 was confirmed by immunoblot analysis. CRISPR/Cas9-mediated ALDH1L1 gene editing in RT4 cells was performed by Synthego Corporation (Redwood city, CA, USA). After electroporating of sgRNA into cells, genomic DNA was isolated, amplified, and assessed by Sanger sequencing. The Sanger trace data were analyzed using Synthego Inference of CRISPR Edits software (Synthego ICE). The sgRNA-edited cell pools were cultured in complete medium.

### 4.3. Western Blot Assays

To confirm ALDH1L1 knockdown efficiency in shRNA and CRISPR clones, we performed Western blot analysis. Cells were lysed in RIPA buffer (Thermo Fisher Scientific) containing protease inhibitors (Thermo Fisher Scientific) and phosphatase inhibitors cocktails (Thermo Fisher Scientific) and quantified using BCA assay. Samples containing equal amounts of total protein were subjected to SDS-PAGE on 8–16% precast Criterion gels ( BioRad, Hercules, CA, USA) followed by Western blotting on PVDF membranes (Millipore, Burlington, MA, USA). Membranes were blocked in TBST buffer with 3% dry milk followed by incubation with in-house anti-ALDH1L1 antibody (1:8000) overnight and then with horseradish peroxidase-conjugated secondary antibody (Cytiva, Marlborough, MA, USA). The ALDH1L1-specific band was detected using chemiluminescent substrate (Millipore) and visualized by an Odyssey FC Imaging System (LI-COR Biosciences, Lincoln, NE, USA). The protein bands were quantified using an Image Studio Lite Software (LI-COR Biosciences).

### 4.4. Immunofluorescence Staining

Cells grown in chamber slides were fixed with 4% paraformaldehyde (Invitrogen, Waltham, MA, USA) for 15 min. Fixed cells were permeabilized with 1% Triton X-100 in PSB for 5 min and incubated overnight with ALDH1L1 in-house polyclonal antibody (1:1000). After 1 h incubation with secondary antibody (donkey anti-rabbit IgG, Alexa Fluor^TM^ 568, Invitrogen) coverslips were mounted with Prolong^TM^ diamond antifade mountant with DAPI (Thermo Fisher Scientific). Images were taken on an Olympus FV10i confocal microscope at 40× magnification. Fiji-Image J software (NIH, Bethesda, MD, USA) was used to quantify immunofluorescence intensity.

### 4.5. Metabolite Extraction

Metabolites were extracted from cells using previously described methods [65]. Briefly, RT4 cells were grown in 10 cm dishes until they reached ~70–80% confluency. Dishes were then placed on ice, cells were washed twice with 10 mL of ice-cold PBS and quenched with 1 mL of ice-cold acetonitrile. After 750 µL of ice-cold water was added to dishes, the cells were dislodged by scraping, and cell suspensions were transferred to 15 mL conical tubes. The procedure was repeated to collect residual cells from the dishes. The two batches were combined to yield a total of 3.5 mL of extract per sample. To ensure complete cell lysis, the extracts were freeze–thawed and then clarified by centrifugation at 16,000× *g* for 10 min at 4 °C. Supernatants were dried in speed-vac and reconstituted in 95:5 water:methanol proportionally to each sample’s protein concentration. A quality control study pool (QCSP) was created by mixing 10 μL of each sample. LC-MS grade water was used to create method blanks. 

### 4.6. Ultra High Performance Liquid Chromatography-High Resolution Mass Spectrometry (UHPLC-HRMS) Analysis

Metabolomics data were acquired with a Vanquish UHPLC system coupled to a Q Exactive™ HF-X Hybrid Quadrupole-Orbitrap Mass Spectrometer (Thermo Fisher Scientific) using previously described methods [7,65,66,67,68]. Cell samples were randomized and injected onto the UHPLC-HRMS platform with QCSP and blank injections occurring every 6 samples. Separation of metabolites was performed using a HSS T3 C18 column (2.1 × 100 mm, 1.7 µm, Waters Corporation) at 50 °C with binary mobile phase of water (A) and methanol (B), each containing 0.1% formic acid (*v*/*v*). The UHPLC linear gradient started from 2% B, and increased to 100% B in 16 min, then held for 4 min, with a flow rate of 400 µL/min. The untargeted data were acquired from 70 to 1050 *m*/*z* using data-dependent acquisition mode. Peak picking, alignment, and normalization was performed using Progenesis QI (version 2.1, Waters Corporation, Milford, MA, USA). QCSP and method blanks were analyzed after every six study samples to evaluate instrument stability and performance throughout data acquisition. Background removal was performed by filtering out peaks with a higher average abundance in the blank injections as compared to the QCSP injections. Peaks were then normalized in Progenesis QI using the “normalize to all” feature [69]. Principal component analysis (PCA) and orthogonal partial least squares-discriminant analysis (OPLS-DA) were performed using SIMCA 16 (Umetrics, Umeå, Sweden). Data quality was assessed by visualizing the clustering and centering of QCSP injections with the study samples in PCA plots [70]. Strong OPLS-DA models were defined as having R2X, R2Y, and Q2 > 0.5.

### 4.7. Metabolite Identification/Annotation and Metabolite Analysis

Identification and annotation of peaks to metabolites was performed by matching to an in-house reference standard RT, Mass, MS/MS library of over 2400 compounds run on the UHPLC-HRMS platform, or to public databases (NIST, METLIN, HMDB). Metabolite assignments were based on matches of peaks to exact mass (MS, <5 ppm), MS/MS fragmentation pattern (similarity score > 30%), isotopic ion pattern (similarity score > 90%), or retention time (RT, for in-house library standards only, ±0.5 min). OL1 refers to an in-house library match by MS, MS/MS, and RT; OL2a refers to an in-house library match by MS and RT; OL2b refers to an in-house library match by MS and MS/MS; PDa refers to a public database match by MS and experimental MS/MS (NIST or METLIN); PDb refers to a public database match by MS and theoretical MS/MS (HMDB); PDc refers to a public database match by MS and isotopic similarity; PDd refers to a public database match by MS only. Metabolites matched at the OL1 and OL2a level were used for analytes conducted in MetaboAnalyst 5.0 [26]. The names given for each match are based on the names of the reference standards run on our UHPLC-HRMS platform or the names provided in public databases. This method does not necessarily differentiate between some isomeric forms such as D and L enantiomers. Network visualization was performed using the Metscape plug-in for Cytoscape [71]. Fold changes were calculated using median peak abundance values and *p*-values were calculated using Student’s t-test. Pathway analysis was performed using the Enrichment Analysis module of MetaboAnalyst 5.0 [26]. Only metabolites matched to the in-house library at a level of OL1 and OL2a with an FDR-corrected *p* < 0.05 in all knockouts vs. wildtype samples were used for the analysis. Pathway analysis was performed separately for increased or decreased metabolites in the knockout vs. wildtype comparison.

## 5. Conclusions

Overall, though our study was exploratory, it supports the hypothesis that downregulation of ALDH1L1 in cancer cells strongly affects cellular metabolism, which might provide proliferative and migratory advantage. ALDH1L1 is considered as one of the major regulators of folate metabolism, the pathway which role in cancer was known for decades [72]. Relevant to the present study, low serum folate was associated with an increased risk of urothelial cell carcinomas of the bladder, particularly its aggressive forms [73]. Importance of folate metabolism in bladder cancer was further suggested by a study which demonstrated overexpression of folate enzyme MTHFD1L in muscle invasive bladder cancer tissues [74]. Of note, antifolate methotrexate is used, in combination with other drugs, for the treatment of bladder cancer [75]. Future studies should test the role of ALDH1L1 in bladder cancer using animal models, as well as the responsiveness of ALDH1L1-proficient versus deficient tumors to chemotherapy.

## Data Availability

All data are included in the manuscript and its Supplementary Materials.

## Supplementary Materials

The following supporting information can be downloaded at: https://www.mdpi.com/article/10.3390/molecules27238394/s1, Figure S1: PCA and sPLSDA analyses of lower number of metabolites identified with higher degrees of confidence (OL1/OL2a or LO1/OL2a/OL2b); Figure S2: Metabolomes comparison between RT4 cells and all clones combined into one group; Figure S3: Jittered boxplot showing the top significant metabolites (OL1 and OL2a ontology levels) selected from the Volcano plots generated in the three-group comparison; Figure S4: Volcano plots of pairwise comparisons between groups with different levels of ALDH1L1 expression (high, medium, and low/undetectable) using OL1 and OL2a metabolites; Table S1: OPLS-DA model statistics for comparisons of wild type RT4 cells (WT) and each ALDH1L1 knockout clone; Table S2: Pairwise comparison between RT4 cells and each ALDH1L1 deficient clone; Table S3: Metabolites with a nominal *p*-value < 0.05 and a fold change >2 selected by volcano plot for WT vs. all ALDH1L1 KO clones combined; File S1: Peak abundances for all identified and annotated metabolties for each sample; File S2: Comparison of metabolites between WT and KO RT4 cells; File S3: Significant (*p* < 0.1) metabolites selected by volcano plots for low vs. high, medium vs. high and low vs. medium ALDH1L1 comparison; File S4: Pathway analysis of WT vs. all KO clones.

## Sample Availability

Generated clones of RT4 cells are available from S. Krupenko upon request.

## References

[1] Krupenko S.A., Oleinik N.V. (2002). 10-Formyltetrahydrofolate Dehydrogenase, One of The Major Folate Enzymes, Is Down-Regulated In Tumor Tissues and Possesses Suppressor Effects On Cancer Cells. Cell Growth Differ..

[2] Krupenko S.A. (2009). Fdh: An Aldehyde Dehydrogenase Fusion Enzyme In Folate Metabolism. Chem. Biol. Interact..

[3] Krupenko S.A., Krupenko N.I. (2019). Loss of Aldh1l1 Folate Enzyme Confers A Selective Metabolic Advantage For Tumor Progression. Chem. Biol. Interact..

[4] Anguera M.C., Field M.S., Perry C., Ghandour H., Chiang E.P., Selhub J., Shane B., Stover P.J. (2006). Regulation of Folate-Mediated One-Carbon Metabolism By 10-Formyltetrahydrofolate Dehydrogenase. J. Biol. Chem..

[5] Hoeferlin L.A., Oleinik N.V., Krupenko N.I., Krupenko S.A. (2011). Activation of P21-Dependent G1/G2 Arrest In The Absence of Dna Damage As An Antiapoptotic Response To Metabolic Stress. Genes Cancer.

[6] Krupenko N.I., Sharma J., Pediaditakis P., Fekry B., Helke K.L., Du X., Sumner S., Krupenko S.A. (2019). Cytosolic 10-Formyltetrahydrofolate Dehydrogenase Regulates Glycine Metabolism In Mouse Liver. Sci. Rep..

[7] Sharma J., Rushing B.R., Hall M.S., Helke K.L., Mcritchie S.L., Krupenko N.I., Sumner S.J., Krupenko S.A. (2022). Sex-Specific Metabolic Effects of Dietary Folate Withdrawal In Wild-Type and Aldh1l1 Knockout Mice. Metabolites.

[8] Krupenko S.A., Cole S.A., Hou R., Haack K., Laston S., Mehta N.R., Comuzzie A.G., Butte N.F., Voruganti V.S. (2022). Genetic Variants In Aldh1l1 and Gldc Influence Serine To Glycine Ratio In Hispanic Children. Am. J. Clin. Nutr..

[9] Shane B., Stokstad E.L. (1985). Vitamin B12-Folate Interrelationships. Annu. Rev. Nutr..

[10] Cooperman J.M., Lopez R. (2002). The Role of Histidine In the Anemia of Folate Deficiency. Exp. Biol. Med..

[11] Beniaminov A.D., Puzanov G.A., Krasnov G.S., Kaluzhny D.N., Kazubskaya T.P., Braga E.A., Kudryavtseva A.V., Melnikova N.V., Dmitriev A.A. (2018). Deep Sequencing Revealed A Cpg Methylation Pattern Associated With Aldh1l1 Suppression In Breast Cancer. Front. Genet..

[12] Tackels-Horne D., Goodman M.D., Williams A.J., Wilson D.J., Eskandari T., Vogt L.M., Boland J.F., Scherf U., Vockley J.G. (2001). Identification of Differentially Expressed Genes In Hepatocellular Carcinoma and Metastatic Liver Tumors By Oligonucleotide Expression Profiling. Cancer.

[13] Hu Z., Yang R., Li L., Mao L., Liu S., Qiao S., Ren G., Hu J. (2020). Validation of Gene Profiles For Analysis of Regional Lymphatic Metastases In Head and Neck Squamous Cell Carcinoma. Front. Mol. Biosci..

[14] Dmitriev A.A., Kashuba V.I., Haraldson K., Senchenko V.N., Pavlova T.V., Kudryavtseva A.V., Anedchenko E.A., Krasnov G.S., Pronina I.V., Loginov V.I. (2012). Genetic and Epigenetic Analysis of Non-Small Cell Lung Cancer With Noti-Microarrays. Epigenetics.

[15] Rodriguez F.J., Giannini C., Asmann Y.W., Sharma M.K., Perry A., Tibbetts K.M., Jenkins R.B., Scheithauer B.W., Anant S., Jenkins S. (2008). Gene Expression Profiling of Nf-1-Associated and Sporadic Pilocytic Astrocytoma Identifies Aldehyde Dehydrogenase 1 Family Member L1 (Aldh1l1) As An Underexpressed Candidate Biomarker In Aggressive Subtypes. J. Neuropathol. Exp. Neurol..

[16] Oleinik N.V., Krupenko N.I., Krupenko S.A. (2011). Epigenetic Silencing of Aldh1l1, A Metabolic Regulator of Cellular Proliferation, In Cancers. Genes Cancer.

[17] Dmitriev A.A., Rudenko E.E., Kudryavtseva A.V., Krasnov G.S., Gordiyuk V.V., Melnikova N.V., Stakhovsky E.O., Kononenko O.A., Pavlova L.S., Kondratieva T.T. (2014). Epigenetic Alterations of Chromosome 3 Revealed By Noti-Microarrays In Clear Cell Renal Cell Carcinoma. Biomed Res. Int..

[18] Senchenko V.N., Kisseljova N.P., Ivanova T.A., Dmitriev A.A., Krasnov G.S., Kudryavtseva A.V., Panasenko G.V., Tsitrin E.B., Lerman M.I., Kisseljov F.L. (2013). Novel Tumor Suppressor Candidates On Chromosome 3 Revealed By Noti-Microarrays In Cervical Cancer. Epigenetics.

[19] Prakasam A., Ghose S., Oleinik N.V., Bethard J.R., Peterson Y.K., Krupenko N.I., Krupenko S.A. (2014). Jnk1/2 Regulate Bid By Direct Phosphorylation At Thr59 In Response To Aldh1l1. Cell Death Dis..

[20] Oleinik N.V., Krupenko S.A. (2003). Ectopic Expression of 10-Formyltetrahydrofolate Dehydrogenase In A549 Cells Induces G(1) Cell Cycle Arrest and Apoptosis. Mol. Cancer Res..

[21] Oleinik N.V., Krupenko N.I., Priest D.G., Krupenko S.A. (2005). Cancer Cells Activate P53 In Response To 10-Formyltetrahydrofolate Dehydrogenase Expression. Biochem. J..

[22] Rodrigues D., Jeronimo C., Henrique R., Belo L., De Lourdes Bastos M., De Pinho P.G., Carvalho M. (2016). Biomarkers In Bladder Cancer: A Metabolomic Approach Using In Vitro and Ex Vivo Model Systems. Int. J. Cancer.

[23] Liu H., Tan Q., Geddie W.R., Jewett M.A., Phillips N., Ke D., Simmons C.A., Sun Y. (2014). Biophysical Characterization of Bladder Cancer Cells With Different Metastatic Potential. Cell Biochem. Biophys..

[24] Iliou A., Panagiotakis A., Giannopoulou A.F., Benaki D., Kosmopoulou M., Velentzas A.D., Tsitsilonis O.E., Papassideri I.S., Voutsinas G.E., Konstantakou E.G. (2020). Malignancy Grade-Dependent Mapping of Metabolic Landscapes In Human Urothelial Bladder Cancer: Identification of Novel, Diagnostic, and Druggable Biomarkers. Int. J. Mol. Sci..

[25] Krupenko S.A., Sharma J. (2021). Is ALDH1L1 Elevated in Lung Cancer? Comment on: Lee, S.-H.; et al. “The Combination of Loss of ALDH1L1 Function and Phenformin Treatment Decreases Tumor Growth in KRAS-Driven Lung Cancer” *Cancers* 2020, *12*, 1382. Cancers.

[26] Pang Z., Chong J., Zhou G., De Lima Morais D.A., Chang L., Barrette M., Gauthier C., Jacques P.E., Li S., Xia J. (2021). Metaboanalyst 5.0: Narrowing The Gap Between Raw Spectra and Functional Insights. Nucleic Acids Res..

[27] Ju H.Q., Lin J.F., Tian T., Xie D., Xu R.H. (2020). Nadph Homeostasis In Cancer: Functions, Mechanisms and Therapeutic Implications. Signal Transduct. Target. Ther..

[28] Chen L., Zhang Z., Hoshino A., Zheng H.D., Morley M., Arany Z., Rabinowitz J.D. (2019). Nadph Production By The Oxidative Pentose-Phosphate Pathway Supports Folate Metabolism. Nat. Metab..

[29] Fan J., Ye J., Kamphorst J.J., Shlomi T., Thompson C.B., Rabinowitz J.D. (2014). Quantitative Flux Analysis Reveals Folate-Dependent Nadph Production. Nature.

[30] Deberardinis R.J., Chandel N.S. (2016). Fundamentals of Cancer Metabolism. Sci. Adv..

[31] Dettmer K., Vogl F.C., Ritter A.P., Zhu W., Nurnberger N., Kreutz M., Oefner P.J., Gronwald W., Gottfried E. (2013). Distinct Metabolic Differences Between Various Human Cancer and Primary Cells. Electrophoresis.

[32] Armitage E.G., Barbas C. (2014). Metabolomics In Cancer Biomarker Discovery: Current Trends and Future Perspectives. J. Pharm. Biomed. Anal..

[33] Sun C., Li T., Song X., Huang L., Zang Q., Xu J., Bi N., Jiao G., Hao Y., Chen Y. (2019). Spatially Resolved Metabolomics To Discover Tumor-Associated Metabolic Alterations. Proc. Natl. Acad. Sci. USA.

[34] Porporato P.E., Filigheddu N., Pedro J.M.B., Kroemer G., Galluzzi L. (2018). Mitochondrial Metabolism and Cancer. Cell Res..

[35] Hanahan D., Weinberg R.A. (2011). Hallmarks of Cancer: The Next Generation. Cell.

[36] Intlekofer A.M., Finley L.W.S. (2019). Metabolic Signatures of Cancer Cells and Stem Cells. Nat. Metab..

[37] Pavlova N.N., Thompson C.B. (2016). The Emerging Hallmarks of Cancer Metabolism. Cell Metab..

[38] Bergers G., Fendt S.M. (2021). The Metabolism of Cancer Cells During Metastasis. Nat. Rev. Cancer.

[39] Fares J., Fares M.Y., Khachfe H.H., Salhab H.A., Fares Y. (2020). Molecular Principles of Metastasis: A Hallmark of Cancer Revisited. Signal Transduct. Target. Ther..

[40] Kaushik A.K., Deberardinis R.J. (2018). Applications of Metabolomics To Study Cancer Metabolism. Biochim. Biophys. Acta Rev. Cancer.

[41] Faubert B., Solmonson A., Deberardinis R.J. (2020). Metabolic Reprogramming and Cancer Progression. Science.

[42] Gaude E., Frezza C. (2016). Tissue-Specific and Convergent Metabolic Transformation of Cancer Correlates With Metastatic Potential and Patient Survival. Nat. Commun..

[43] Hu J., Locasale J.W., Bielas J.H., O’sullivan J., Sheahan K., Cantley L.C., Vander Heiden M.G., Vitkup D. (2013). Heterogeneity of Tumor-Induced Gene Expression Changes In The Human Metabolic Network. Nat. Biotechnol..

[44] Chowdhry S., Zanca C., Rajkumar U., Koga T., Diao Y., Raviram R., Liu F., Turner K., Yang H., Brunk E. (2019). Nad Metabolic Dependency In Cancer Is Shaped By Gene Amplification and Enhancer Remodelling. Nature.

[45] Pupo E., Avanzato D., Middonti E., Bussolino F., Lanzetti L. (2019). Kras-Driven Metabolic Rewiring Reveals Novel Actionable Targets In Cancer. Front. Oncol..

[46] Zahra K., Dey T., Ashish, Mishra S.P., Pandey U. (2020). Pyruvate Kinase M2 and Cancer: The Role of Pkm2 In Promoting Tumorigenesis. Front. Oncol..

[47] Krupenko N.I., Sharma J., Fogle H.M., Pediaditakis P., Strickland K.C., Du X., Helke K.L., Sumner S., Krupenko S.A. (2021). Knockout of Putative Tumor Suppressor Aldh1l1 In Mice Reprograms Metabolism To Accelerate Growth of Tumors In A Diethylnitrosamine (Den) Model of Liver Carcinogenesis. Cancers.

[48] Lee S.H., Jeon Y., Kang J.H., Jang H., Lee H., Kim S.Y. (2020). The Combination of Loss of Aldh1l1 Function and Phenformin Treatment Decreases Tumor Growth In Kras-Driven Lung Cancer. Cancers.

[49] Verma N., Pink M., Boland S., Rettenmeier A.W., Schmitz-Spanke S. (2017). Benzo[A]Pyrene-Induced Metabolic Shift From Glycolysis To Pentose Phosphate Pathway In The Human Bladder Cancer Cell Line Rt4. Sci. Rep..

[50] Conde V.R., Oliveira P.F., Nunes A.R., Rocha C.S., Ramalhosa E., Pereira J.A., Alves M.G., Silva B.M. (2015). The Progression From A Lower To A Higher Invasive Stage of Bladder Cancer Is Associated With Severe Alterations In Glucose and Pyruvate Metabolism. Exp. Cell Res..

[51] Loras A., Martinez-Bisbal M.C., Quintas G., Gil S., Martinez-Manez R., Ruiz-Cerda J.L. (2019). Urinary Metabolic Signatures Detect Recurrences In Non-Muscle Invasive Bladder Cancer. Cancers.

[52] Bansal A., Simon M.C. (2018). Glutathione Metabolism In Cancer Progression and Treatment Resistance. J. Cell Biol..

[53] Moore L.E., Malats N., Rothman N., Real F.X., Kogevinas M., Karami S., Garcia-Closas R., Silverman D., Chanock S., Welch R. (2007). Polymorphisms In One-Carbon Metabolism and Trans-Sulfuration Pathway Genes and Susceptibility To Bladder Cancer. Int. J. Cancer.

[54] Stretch C., Eastman T., Mandal R., Eisner R., Wishart D.S., Mourtzakis M., Prado C.M., Damaraju S., Ball R.O., Greiner R. (2012). Prediction of Skeletal Muscle and Fat Mass In Patients With Advanced Cancer Using A Metabolomic Approach. J. Nutr..

[55] Makarov M.V., Trammell S.A.J., Migaud M.E. (2019). The Chemistry of The Vitamin B3 Metabolome. Biochem. Soc. Trans..

[56] Landgraf B.J., Mccarthy E.L., Booker S.J. (2016). Radical S-Adenosylmethionine Enzymes In Human Health and Disease. Annu. Rev. BioChem..

[57] Mpanga A.Y., Siluk D., Jacyna J., Szerkus O., Wawrzyniak R., Markuszewski M., Matuszewski M., Kaliszan R., Markuszewski M.J. (2018). Targeted Metabolomics In Bladder Cancer: From Analytical Methods Development and Validation Towards Application To Clinical Samples. Anal. Chim. Acta.

[58] Saad A.A., O’connor P.J., Mostafa M.H., Metwalli N.E., Cooper D.P., Margison G.P., Povey A.C. (2006). Bladder Tumor Contains Higher N7-Methylguanine Levels In Dna Than Adjacent Normal Bladder Epithelium. Cancer Epidemiol. Biomark. PRev..

[59] Rinne M.L., He Y., Pachkowski B.F., Nakamura J., Kelley M.R. (2005). N-Methylpurine Dna Glycosylase Overexpression Increases Alkylation Sensitivity By Rapidly Removing Non-Toxic 7-Methylguanine Adducts. Nucleic Acids Res..

[60] Nilov D., Maluchenko N., Kurgina T., Pushkarev S., Lys A., Kutuzov M., Gerasimova N., Feofanov A., Svedas V., Lavrik O. (2020). Molecular Mechanisms of Parp-1 Inhibitor 7-Methylguanine. Int. J. Mol. Sci..

[61] Billson H.A., Harrison K.L., Lees N.P., Hall C.N., Margison G.P., Povey A.C. (2009). Dietary Variables Associated With Dna N7-Methylguanine Levels and O6-Alkylguanine Dna-Alkyltransferase Activity In Human Colorectal Mucosa. Carcinogenesis.

[62] Petrella G., Ciufolini G., Vago R., Cicero D.O. (2020). The Interplay Between Oxidative Phosphorylation and Glycolysis As A Potential Marker of Bladder Cancer Progression. Int. J. Mol. Sci..

[63] Khan Q.A., Pediaditakis P., Malakhau Y., Esmaeilniakooshkghazi A., Ashkavand Z., Sereda V., Krupenko N.I., Krupenko S.A. (2018). Chip E3 Ligase Mediates Proteasomal Degradation of The Proliferation Regulatory Protein Aldh1l1 During The Transition of Nih3t3 Fibroblasts From G0/G1 To S-Phase. PLoS ONE.

[64] Oleinik N.V., Krupenko N.I., Reuland S.N., Krupenko S.A. (2006). Leucovorin-Induced Resistance Against Fdh Growth Suppressor Effects Occurs Through Dhfr Up-Regulation. Biochem. Pharmacol..

[65] Rushing B.R., Schroder M., Sumner S.C.J. (2022). Comparison of Lysis and Detachment Sample Preparation Methods For Cultured Triple-Negative Breast Cancer Cells Using Uhplc-Hrms-Based Metabolomics. Metabolites.

[66] Rushing B.R., Tilley S., Molina S., Schroder M., Sumner S. (2022). Commonalities In Metabolic Reprogramming Between Tobacco Use and Oral Cancer. Int. J. Environ. Res. Public Health.

[67] Li S., Li Y., Rushing B.R., Harris S.E., Mcritchie S.L., Dominguez D., Sumner S.J., Dohlman H.G. (2022). Multi-Omics Analysis of Multiple Glucose-Sensing Receptor Systems In Yeast. Biomolecules.

[68] Li S., Li Y., Rushing B.R., Harris S.E., Mcritchie S.L., Jones J.C., Dominguez D., Sumner S.J., Dohlman H.G. (2021). Multi-Omics Analysis of Glucose-Mediated Signaling By A Moonlighting Gbeta Protein Asc1/Rack1. PLoS Genet..

[69] Valikangas T., Suomi T., Elo L.L. (2018). A Systematic Evaluation of Normalization Methods In Quantitative Label-Free Proteomics. Brief. Bioinform..

[70] Broadhurst D., Goodacre R., Reinke S.N., Kuligowski J., Wilson I.D., Lewis M.R., Dunn W.B. (2018). Guidelines and Considerations For The Use of System Suitability and Quality Control Samples In Mass Spectrometry Assays Applied In Untargeted Clinical Metabolomic Studies. Metabolomics.

[71] Gao J., Tarcea V.G., Karnovsky A., Mirel B.R., Weymouth T.E., Beecher C.W., Cavalcoli J.D., Athey B.D., Omenn G.S., Burant C.F. (2010). Metscape: A Cytoscape Plug-In For Visualizing and Interpreting Metabolomic Data In The Context of Human Metabolic Networks. Bioinformatics.

[72] Strickland K.C., Krupenko N.I., Krupenko S.A. (2013). Molecular Mechanisms Underlying The Potentially Adverse Effects of Folate. Clin. Chem. Lab. Med..

[73] Vrieling A., Bueno-De-Mesquita H.B., Ros M.M., Kampman E., Aben K.K., Buchner F.L., Jansen E.H., Roswall N., Tjonneland A., Boutron-Ruault M.C. (2019). One-Carbon Metabolism Biomarkers and Risk of Urothelial Cell Carcinoma In The European Prospective Investigation Into Cancer and Nutrition. Int. J. Cancer.

[74] Eich M.L., Rodriguez Pena M.D.C., Chandrashekar D.S., Chaux A., Agarwal S., Gordetsky J.B., Ferguson J.E., Sonpavde G.P., Netto G.J., Varambally S. (2019). Expression and Role of Methylenetetrahydrofolate Dehydrogenase 1 Like (Mthfd1l) In Bladder Cancer. Transl. Oncol..

[75] Kamat A.M., Hahn N.M., Efstathiou J.A., Lerner S.P., Malmstrom P.U., Choi W., Guo C.C., Lotan Y., Kassouf W. (2016). Bladder Cancer. Lancet.

