# Supplementary material for: Exploratory Metabolomics Underscores the Folate Enzyme ALDH1L1 as a Regulator of Glycine and Methylation Reactions
Source: Molecules. 2022 Dec 1;27(23):8394. doi: 10.3390/molecules27238394 (PMC9740053; doi:10.3390/molecules27238394)
Supplement: Supplementary file 1 [file molecules-27-08394-s001.zip › Supplementary Materials.pdf]

SUPPLEMENTARY MATERIAL

1. Supplementary Table S1
2. Supplementary Table S2
3. Supplementary Table S3
4. Supplementary Figure S1
5. Supplementary Figure S2
6. Supplementary Figure S3
7. Supplementary Figure S4
8. Supplementary File S1
9. Supplementary File S2
10. Supplementary File S3
11. Supplementary File S4

Supplementary Table S1. OPLS-DA model statistics for comparisons of wild type RT4 cells (WT) and each ADH1L1 knockout clone*.

Comparison	R2X	R2Y	Q2
WT vs L1-CR	0.805	0.991	0.874
WT vs 506	0.812	0.997	0.988
WT vs 572	0.655	0.957	0.759

*Scores were calculated in SIMCA 16. Data was UV scaled.

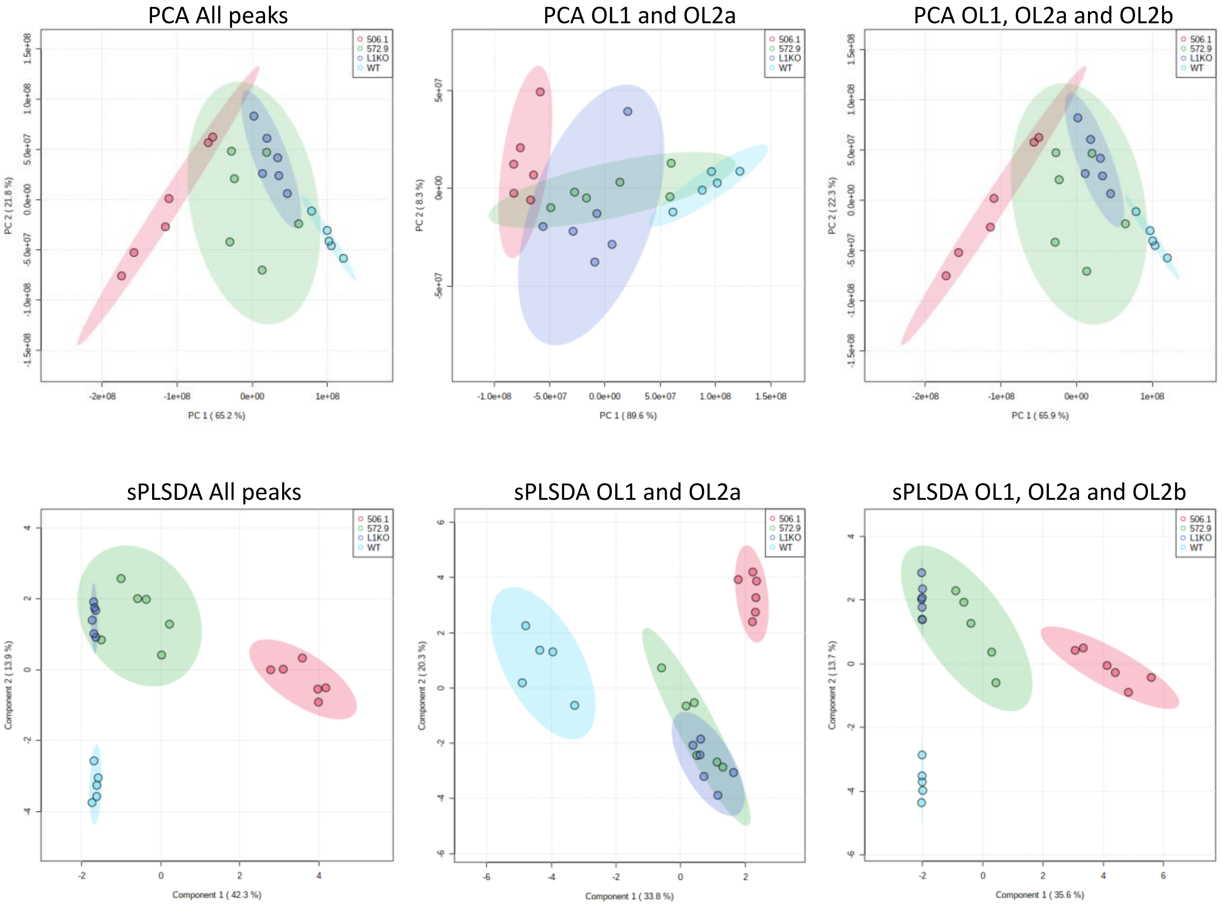

Supplementary Figure S1. PCA and sPLSDA analyses of lower number of metabolites identified with higher degrees of confidence (OL1/OL2a or OL1/OL2a/OL2b).

QC samples - including quality control study pools (QCSPs) and method blanks - were run before and after every 6 study samples. The blanks were used to filter background while the QCSPs were used to align peaks and assess instrument variability across the analysis.

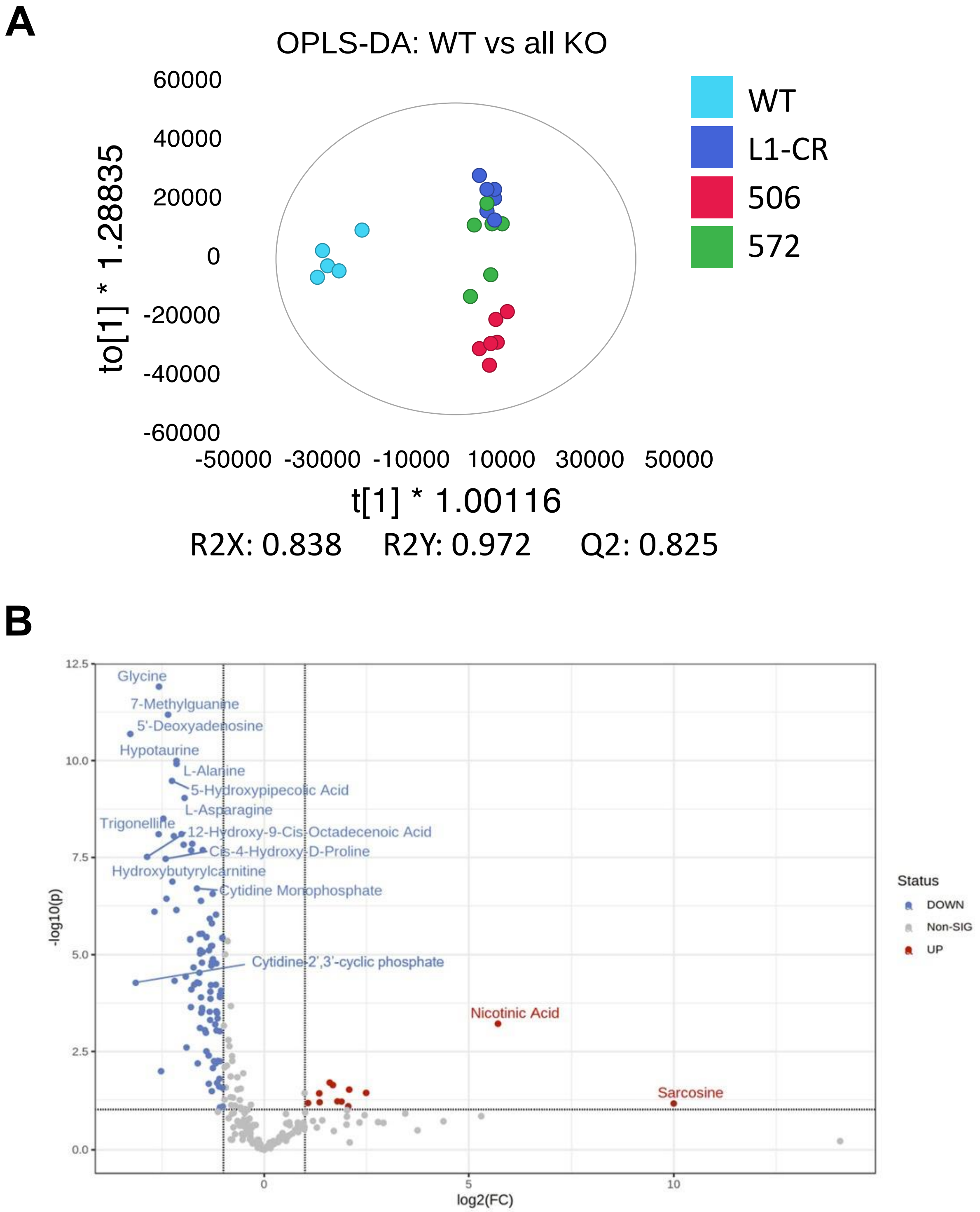

Supplementary Figure S2. Metabolomes comparison between RT4 cells and all clones combined into one group. (A) OPLS-DA between RT4 cells and ALDH1L1 KO cells (R2X: 0.838, R2Y: 0.972, Q2: 0.825). (B) Volcano plots determined top OL1 and OL2a metabolites separating RT4 cells and the KO group. Fold change (FC) was calculated as the mean of KO divided by mean of WT.

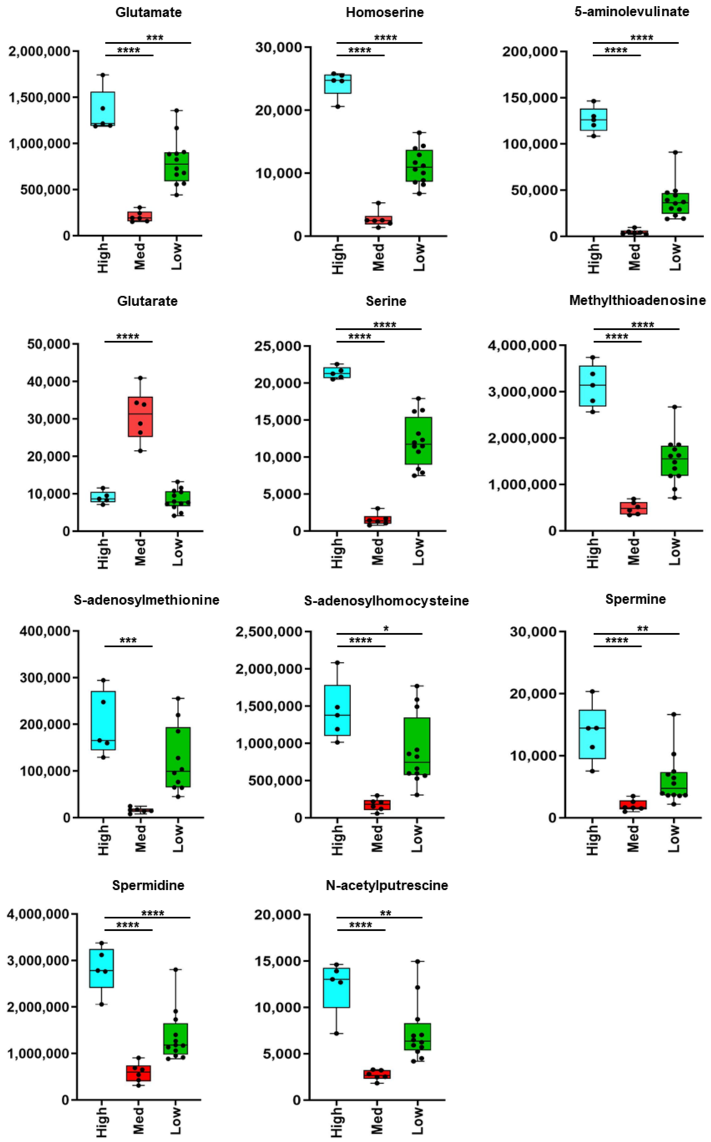

Supplementary Figure S3. Jittered boxplot showing the top significant metabolites (OL1 and OL2a ontology levels) selected from the Volcano plots generated in the three-group comparison. Y axis values represent normalized peak area counts from the MS analysis. The comparison demonstrates non-linear relationships with ALDH1L1 expression. Statistical analysis was done using ANOVA with Dunnett's multiple comparison test. ****q<0.0001; ***q<0.001; **q<0.01; *q<0.05

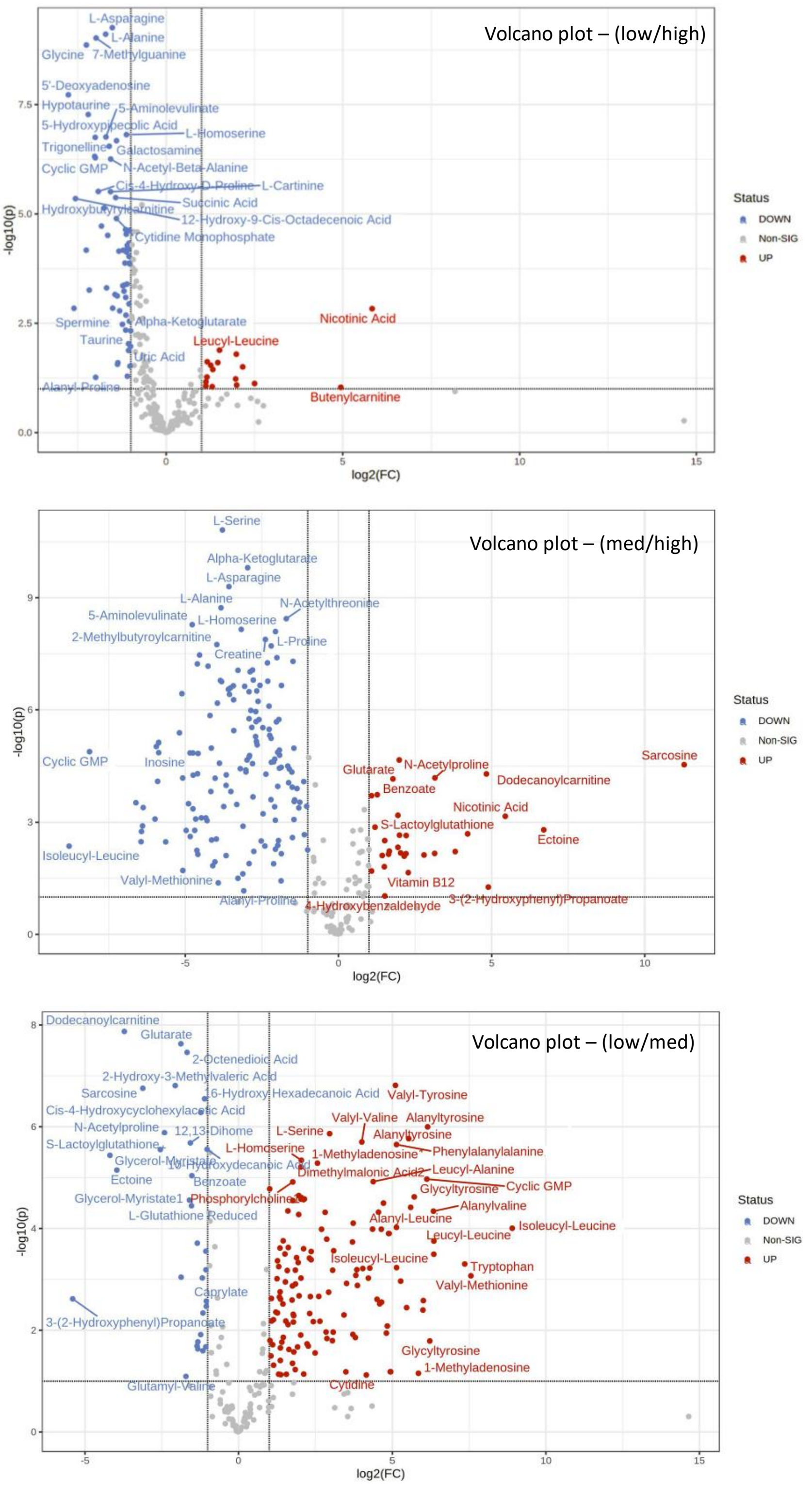

Supplementary Figure S4. Volcano plots of pairwise comparisons between groups with different levels of ALDH1L1 expression (high, medium and low/undetectable) using OL1 and OL2a metabolites.
